# Supplementary material for: Single-cell profiling identifies pre-existing CD19-negative subclones in a B-ALL patient with CD19-negative relapse after CAR-T therapy
Source: Nat Commun. 2021 Feb 8;12:865. doi: 10.1038/s41467-021-21168-6 (PMC7870924; doi:10.1038/s41467-021-21168-6)
Supplement: Supplementary file 1 — Supplementary Information [file 41467_2021_21168_MOESM1_ESM.pdf]

Supplementary information for

Single-cell profiling identifies pre-existing CD19-negative subclones in a B-ALL patient with CD19-negative relapse after CAR-T therapy.

This PDF includes:

- **Supplementary Table 1 to 2**
- **Supplementary Figures 1 to 11**

Supplementary Table

Supplementary Table 1: Oligonucleotide sequences.
All oligonucleotides were synthesized by Integrated DNA Technologies (IDT) Company. For HTO, the specific index sequences are highlighted in red and (*) indicates a phosphorothioate bond. For BC primers, 10X cell identifier barcodes are indicated in green.

	**Sequences of HTO**
**HTO-A**	GTGACTGGAGTTCAGACGTGTGCTCTTCCGATCT AAGTATCGTTTCGCA CCCATATAAGA*A*A
**HTO-B**	GTGACTGGAGTTCAGACGTGTGCTCTTCCGATCT CTTTGTCTTTGTGAG CCCATATAAGA*A*A
**HTO-C**	GTGACTGGAGTTCAGACGTGTGCTCTTCCGATCT CTTGCCGCATGTCAT CCCATATAAGA*A*A
**HTO-D**	GTGACTGGAGTTCAGACGTGTGCTCTTCCGATCT AAAGCATTCTTCACG CCCATATAAGA*A*A
	**Primers for PCR/nested PCR**
**P12**	CTA CAC GAC GCT CTT CCG ATC T
**P10**	CCTGGGAGAGCTGGTCCTGCC
**P14**	CCTCCTCACTGTCAGGTTCCT
**P13**	CGTACTGGTTCTGGGGCCCGC
**P3**	AGAGTCTGACCACCATGCC
**P4**	TTGGGGACTTGAGGAGATCC
**P8**	CCCTCCACATTGACTGTCC
**P9**	GGCCGAGCAGTGATCTCCAGG
**P33**	GAGGCTCTGGTTCAGGCTGT
	**BC primers for cell backtracking**
**BC 2**	GATCTCACCACTTCCTTGACC
**BC 3**	GATCTGTGCGGTTCGAGGTAG
**BC 4**	GATCTCTAGTGACAATGGTCT
**BC 6**	GATCTGACACGCAGTCCGGTC
**BC 7**	GATCTGTTACAGGTTACAGAA
**BC 8**	GATCTCCTCAGTGTGATGATA
**BC 11**	GATCTCCGTGGACAGCTGGCT
**BC 12**	GATCTGTTAAGCTCTAACCGA
**BC 16**	GATCTAGATCTGGTGAGGCTA
**BC 17**	GATCTGAATAAGGTGCACTTA
**BC 18**	GATCTGGACGTCGTAAACACA
**BC 19**	GATCTTACACGAAGTACGCCC
**BC 20**	GATCTATTATCCTCAGATAAG
**BC 21**	GATCTATCATCTGTGGACGAT
**BC 22**	GATCTGCATGCGGTCGAAAGC
**BC 32**	GATCTGAACATCAGTGCCAGA
**BC 33**	GATCTCACACCTGTCGAACAG
**BC 34**	GATCTGACGTGCTCGGAAATA
**BC 35**	GATCTGCTTGAACAATGGACG
**BC 38**	GATCTCGGAGTCGTATGCTTG

Supplementary Table 2: Antibodies used for flow cytometry.

Antigen	Fluorochrome	Clone	Manufacturer	Catalogue number	Lot number	Final dilution
**CD19**	PC7	J3-119	Beckman Coulter	IM3628	200148	1/20
**CD45**	Krome Orange (KrO)	J33	Beckman Coulter	B36294	200070	1/20
**CD34**	ECD	581	Beckman Coulter	B49202	200025	1/20
**CD10**	FITC	ALB1	Beckman Coulter	A07759	200043	1/20
**CD38**	APC	HB7	BD	345807	9113909	1/20
**CD58**	PC5	AICD58	Beckman Coulter	IM3702	200019	1/20
**CD66c**	PE	KORSA354	Beckman Coulter	IM2357U	200052	1/20
**CD123**	PE	9F5	BD	340545	8087759	1/20
**CD3**	PE	HIT3a	BD	555340	4066560	1/5
**CD19**	APC	HIB19	Biolegend	302212	B295177	1/20

Supplementary Figures

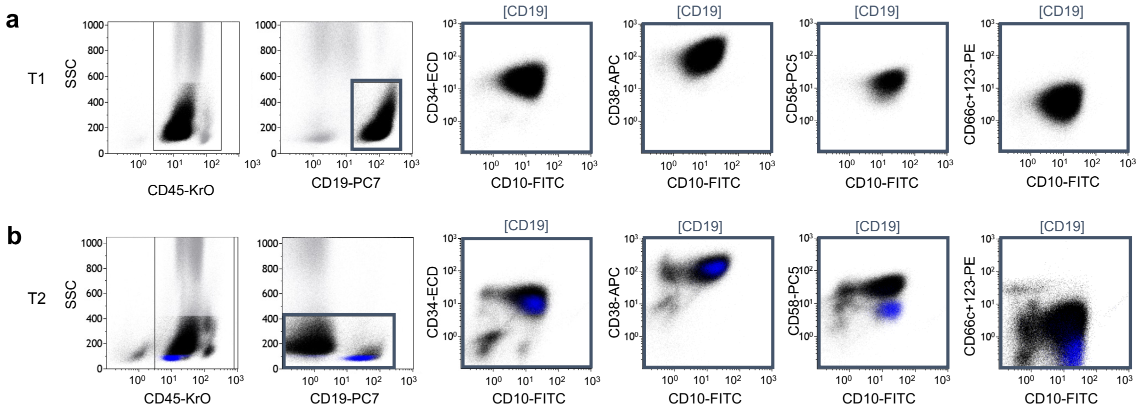

Supplementary Figure 1: Flow cytometry immunophenotyping of leukemic blasts.
Cells were stained with CD45-KrO, CD19-PC7, CD34-ECD, CD10-FITC, CD38-APC, CD58-PC5, CD66c-PE and CD123-PE. Dot plots show the expression of CD45, CD19, CD10, CD34, CD38, CD58 and CD66c+CD123 on blasts cells before CAR-T treatment (**a**) and at relapse (**b**), three months after. (**a**) At T1, blasts cells (in black) are $CD45^{dim}$ and $CD19^{pos}$. Gating on $CD19^{pos}$ cells, blasts are $CD10^{pos}$, $CD34^{pos}$, $CD38^{pos}$, $CD58^{pos}$ and $CD66+CD123^{pos}$. These markers are used as measurable residual disease (MRD) markers. (**b**) At T2, blasts cells are $CD45^{dim}$ but $CD19^{neg}$. They still express the same markers of MRD allowing us to differentiate them from physiological immature B-cells (in blue, around 4% of live cells).

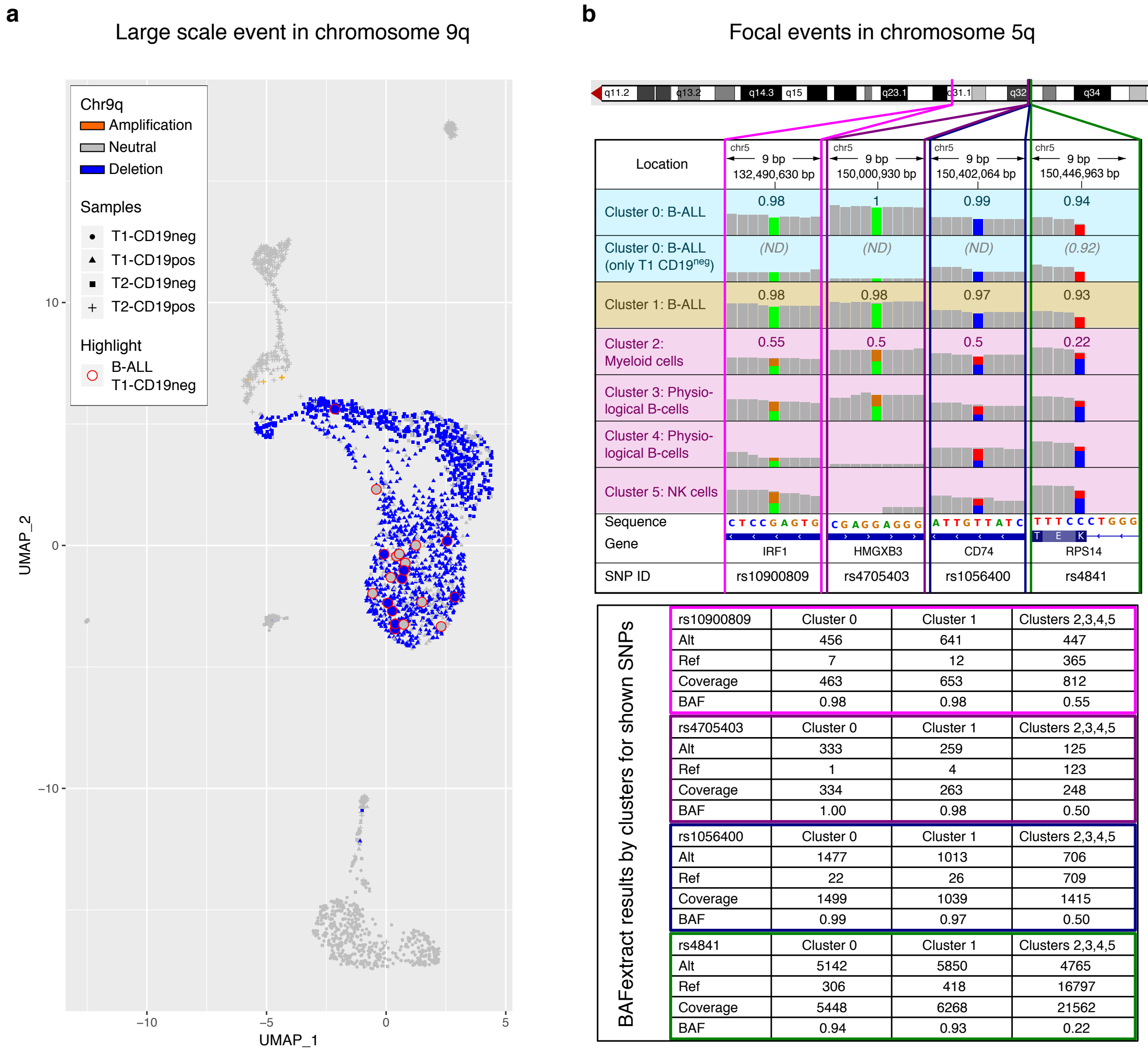

rs10900809	Cluster 0	Cluster 1	Clusters 2,3,4,5
Alt	456	641	447
Ref	7	12	365
Coverage	463	653	812
BAF	0.98	0.98	0.55
rs4705403	Cluster 0	Cluster 1	Clusters 2,3,4,5
Alt	333	259	125
Ref	1	4	123
Coverage	334	263	248
BAF	1.00	0.98	0.50
rs1056400	Cluster 0	Cluster 1	Clusters 2,3,4,5
Alt	1477	1013	706
Ref	22	26	709
Coverage	1499	1039	1415
BAF	0.99	0.97	0.50
rs4841	Cluster 0	Cluster 1	Clusters 2,3,4,5
Alt	5142	5850	4765
Ref	306	418	16797
Coverage	5448	6268	21562
BAF	0.94	0.93	0.22

Supplementary Figure 2: Analysis of allelic imbalance using scRNAseq data.
(**a**) UMAP plot displaying large-scale events detected for chromosome 9q by using CaSpER tool. Cells with normal copy number, deletion or amplification of chromosome 9q are respectively colored in grey, blue and orange. The 20 T1 CD19^{neg} cells within B-ALL clusters are represented by larger dots circled in red. In the B-ALL clusters, the majority of the cells appears in blue, thus this CaSpER analysis predicts that one chromosome 9q is deleted in B-ALL cells. (**b**) BAF analysis of SNPs located in chromosome 5q. In the upper panel, IGV (integrative genomics viewer) screenshots show individual SNPs. BAF values of these SNPs in clusters 0, 1 and in physiological cells' clusters (2, 3, 4 & 5) are indicated (clusters are numbered as mentioned in figure 1). Two independent BAF analysis were performed for Cluster 0. The first analysis labelled 'Cluster 0: B-ALL' comprises all cells from cluster 0. The second analysis labelled 'Cluster 0: B-ALL (only T1 CD19^{neg} cells)' comprises only T1 CD19^{neg} cells from cluster 0. Thus for those T1 CD19^{neg} B-ALL cells, BAF were calculated independently. Yet, a BAF value was obtained only for *RPS14*; ND, indicates that the BAF value could not be determined because of a low number of sequencing reads. BAF close to 1 indicates absence of one of the two alleles, while a BAF close to 0.5 indicates the equal presence of both alleles. Green, red, orange and blue bar colors

correspond to A, T, G and C nucleotides respectively. In the bottom panel, the table displays numbers of reads which maps to either the reference (Ref) or alternative (Alt) allele at each SNP. Those analyses were focused on chromosomes 9q & 5q as clinical cytogenetic analyses of T1 & T2 B-ALL samples detected alterations within these chromosomes.

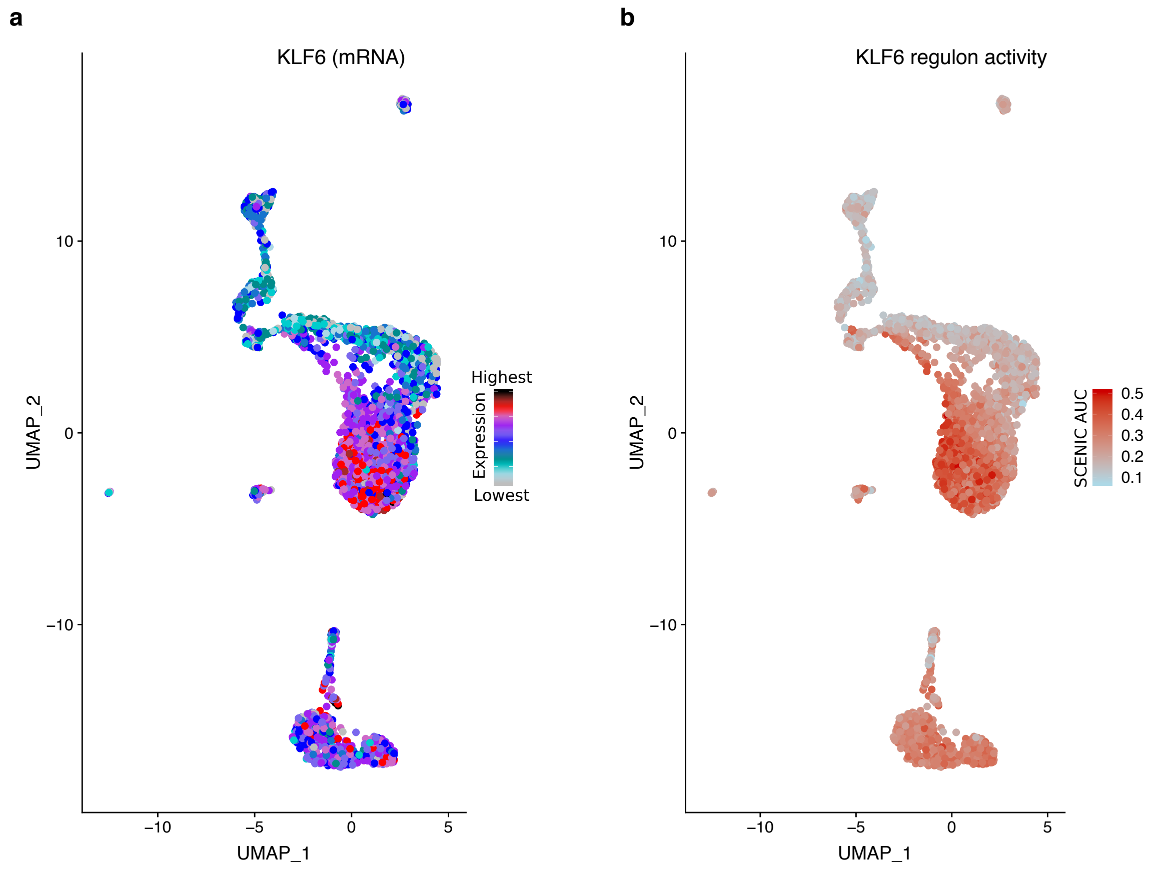

Supplementary Figure 3: KLF6 expression in tumoral clusters.
UMAP visualization of KLF6 mRNA expression (**a**) and regulon activity (**b**) in the 6 main clusters. In cluster 1, down-regulation of KLF6 comes along a decrease of its activity as assessed by its regulon activity using SCENIC.

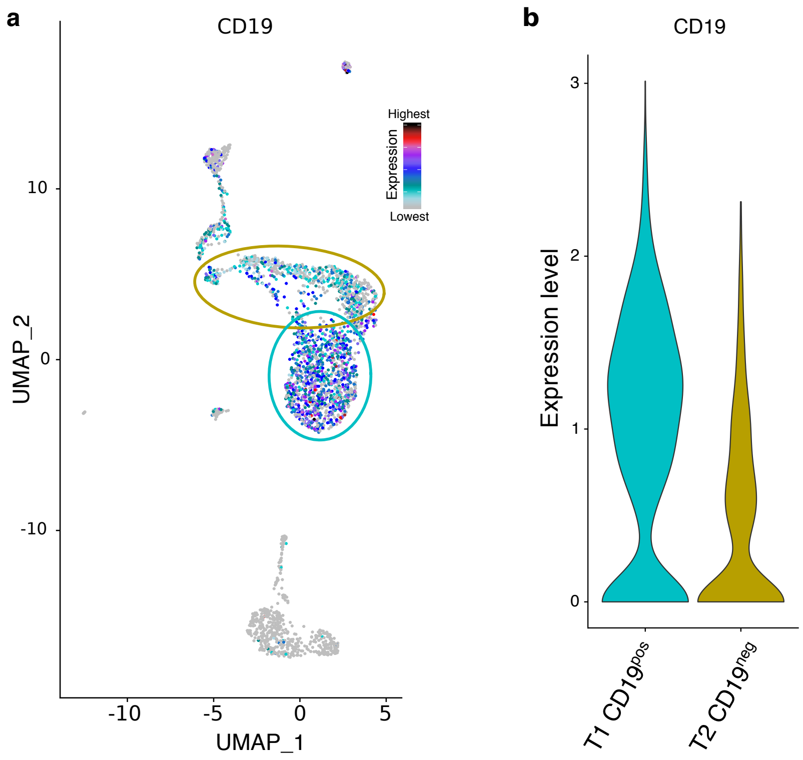

c

5'3' Frame 3

```
aggcc cct gcc tgc ccc agc atc ccc tgc gcg aag ctg ggt gcc ccg gag agt ctg acc acc
   A   P   A   C   P   S   I   P   C   A   K   L   G   A   P   E   S   L   T   T
atg cca cct cct cgc ctc ctc ttc ttc ctc ctc ttc ctc acc ccc atg gaa gtc agg ccc
 M   P   P   P   R   L   L   F   F   L   L   F   L   T   P   M   E   V   R   P
gag gaa cct cta gtg gtg aag gtg gaa gag gga gat aac gct gtg ctg cag tgc ctc aag
 E   E   P   L   V   V   K   V   E   E   G   D   N   A   V   L   Q   C   L   K
ggg acc tca gat ggc ccc act cag cag ctg acc tgg tct cgg gag tcc ccg ctt aaa ccc
 G   T   S   D   G   P   T   Q   Q   L   T   W   S   R   E   S   P   L   K   P
ttc tta aaa ctc agc ctg ggg ctg cca ggc ctg gga atc cac atg agg ccc ctg gcc atc
 F   L   K   L   S   L   G   L   P   G   L   G   I   H   M   R   P   L   A   I
tgg ctt ttc atc ttc aac gtc tct caa cag atg ggg ggc ttc tac ctg tgc cag ccg ggg
 W   L   F   I   F   N   V   S   Q   Q   M   G   G   F   Y   L   C   Q   P   G
ccc ccc tct gag aag gcc tgg cag cct ggc tgg aca gtc aat gtg gag ggc agc ggt gag
 P   P   S   E   K   A   W   Q   P   G   W   T   V   N   V   E   G   S   G   E
ggc cgg gct ggg gca ggg gca gga gga gag aag gga ggc cac cat gga cag aag agg tcc
 G   R   A   G   A   G   A   G   G   E   K   G   G   H   H   G   Q   K   R   S
gcg gcc aca atg gag ctg gag aga ggg gct gga ggg att gag ggc gaa act cgg agc tag
 A   A   T   M   E   L   E   R   G   A   G   G   I   E   G   E   T   R   S  STOP
```

Transcript codons
PROTEIN AMINO ACIDS (**WT extracellular domain**)

nnn 5’ UTR
nnn retained intron 2
nnn exon 1
nnn exon 2

Open reading frame

Supplementary Figure 4: Analysis of *CD19* transcript.
UMAP visualization (**a**) and violin plot (**b**) of *CD19* mRNA expression in T1-CD19^{pos} cells (blue) and T2-CD19^{neg} cells (gold). Here mostly the 5’ region of *CD19* mRNA is captured since we used 5’ 10X genomic approach. **(c)** Non-functional *CD19* mRNA isoform is expressed in leukemic cells. Sanger sequencing of RT-PCR products (Fig. 1e) reveals intron 2 retention. *CD19* transcript codons and their corresponding protein amino acids from 5' UTR region to retained intron 2 (purple) which contained a TAG stop codon leading to a truncated CD19 protein.

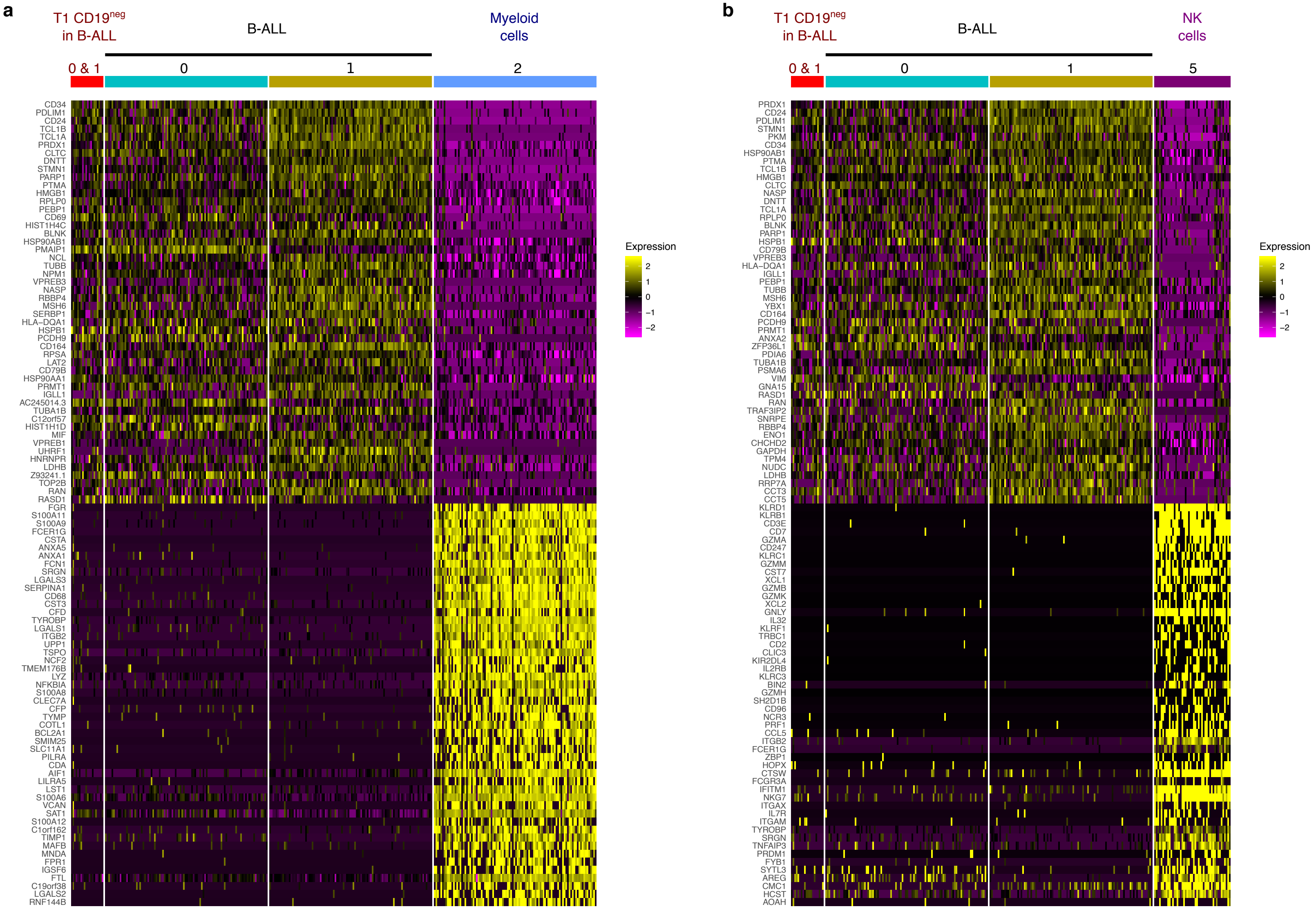

Supplementary Figure 5: Differential gene expression between tumoral and physiological cells. Heatmaps showing normalized and scaled expression level of the top 100 differentially expressed genes (50 up- and 50 down-regulated genes) between tumoral cells (clusters 0 & 1 excluding T1-CD19^{neg} cells) and (**a**) myeloid cells (cluster 2) or (**b**) NK cells (cluster 5). Gene expression profiles of T1-CD19^{neg} cells found in clusters 0 & 1 are shown independently from the other B-ALL cells. Clusters 0, 1 & 2 were down-sampled to 100 cells for a better readability

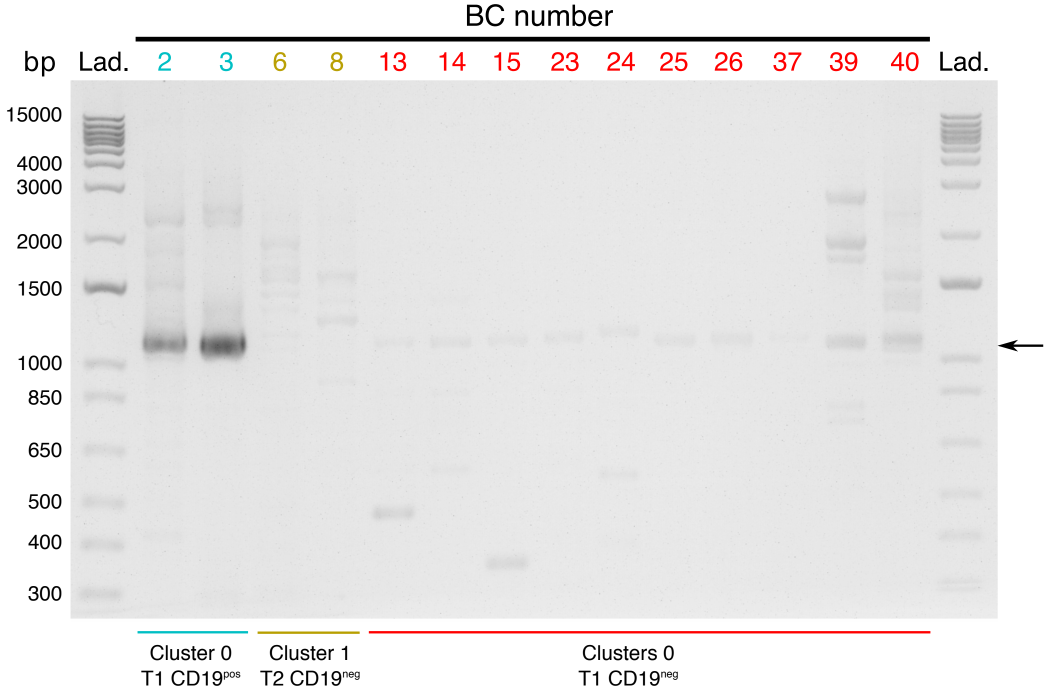

Supplementary Figure 6: Backtracking of CD19^{neg} clones.

Agarose gel of CD19 cDNA amplified by nested-PCR as described in figure 2e. Lanes are labelled according to the cell-BC number for which the sample of origin is indicated at the bottom of the gel. Here are shown putative T1-CD19^{neg} B-ALL clones (cells with BC number: 13, 14, 15, 23, 24, 25, 26, 37, 39 and 40) that were considered ambiguous since a faint band corresponding to *CD19* mRNA (indicated by an arrow) was detected. However, intensities of this band are much lower than in CD19^{pos} cells (BC number 2 and 3), thus we conclude that in accordance with the assignment of FACS-sorting, these T1-CD19neg B-ALL clones are truly CD19-negative.

Cells with BC number 2 and 3 are CD19-positive, while cells with BC number 6 and 8 are CD19-negative cells.

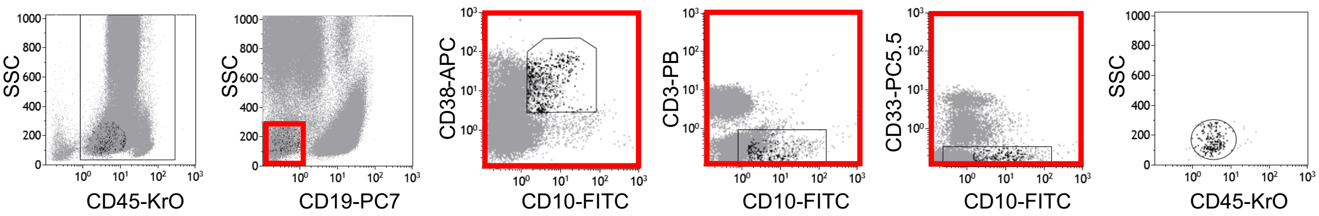

Supplementary Figure 7: FACS analysis of CD19^{neg} cells before CAR-T treatment.

T1 cells were first gated on SSC/CD45 to eliminate cellular debris. Then, CD19^{neg} cells were selected and analyzed according to the positivity of MRD markers (CD38^{pos}, CD10^{neg}) and the negativity of lymphoid T-cell (CD3) and myeloid cell (CD33) markers. The boolean gate shows the cells with the appropriate phenotype.

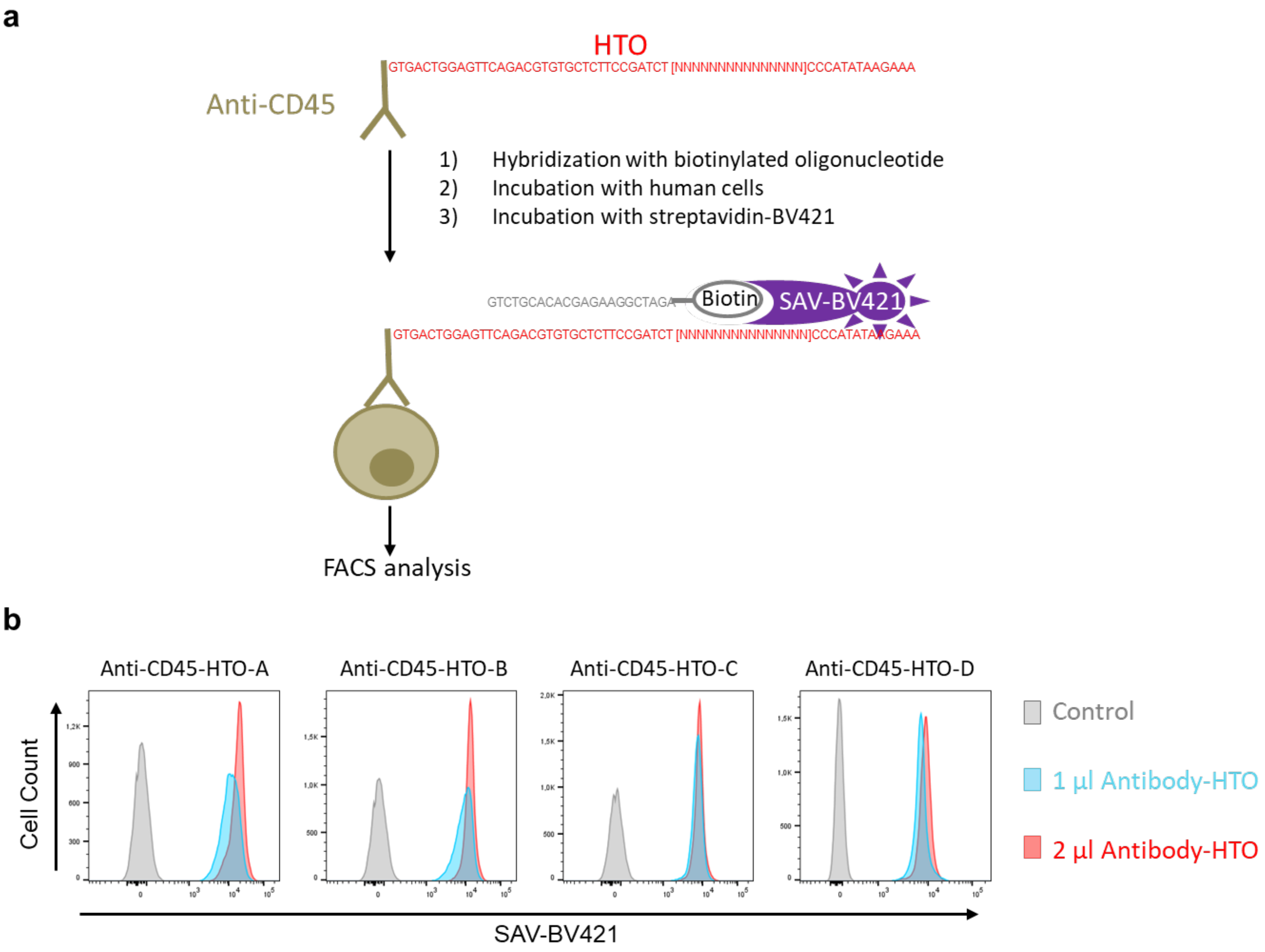

Supplementary Figure 8: Functional validation of conjugated anti-CD45-HTO antibodies

(**a**) Validation strategy. HTO-Antibodies were first hybridized with complementary biotinylated oligonucleotide (Biotin-AG ATC GGA AGA GCA CAC GTC TG). Then, they were incubated with human Jurkat cell line (1.10^6 cells) in 100 µl of FACS buffer (PBS, 2 % FCS, 1 mM EDTA) (30 min at 4°C). After a wash in FACS buffer, 10 µg of streptavidine (SAV) coupled to BV421 (BD Biosciences) were added and samples were further incubated 10 min at 4°C, then they were washed with FACS buffer and finally analyzed on a CANTO II cytometer (BD). Data were analyzed using FlowJo (10.5.3) software. (**b**) Typical histograms obtained for anti-CD45 antibodies coupled to HTO-A, HTO-B, HTO-C and HTO-D. The assays were performed with 1 µl or 2 µl of Antibody-HTO stock solution (estimated concentration of 0,5 µg/µl). Control corresponds to the assay performed in absence of antibody.

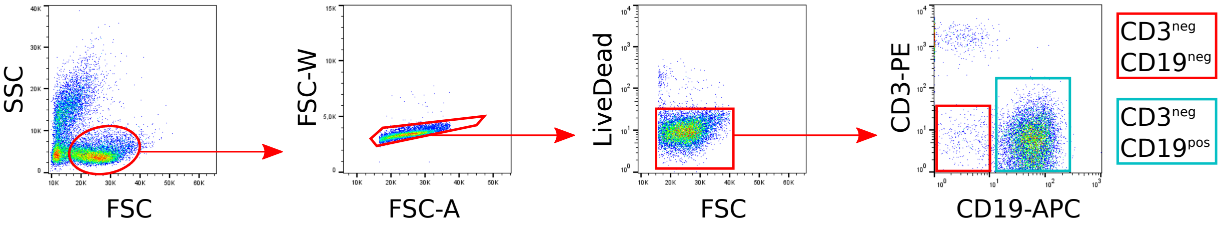

Supplementary Figure 9: The gating strategy used for cell sorting.
Forward versus Side Scatter (FSC vs SSC) was used to include lymphocytes and exclude any red blood cells, apoptotic cells, or debris. Also, a forward scatter width (FSC-W) versus forward scatter area (FSC-A) density plot was used to exclude doublet cells. Then, LIVE/DEAD™ Fixable Aqua Dead Cell Stain Kit (Invitrogen) was used to exclude dead cells. Finally, live cells were sorted according to CD3 and CD19 expression.

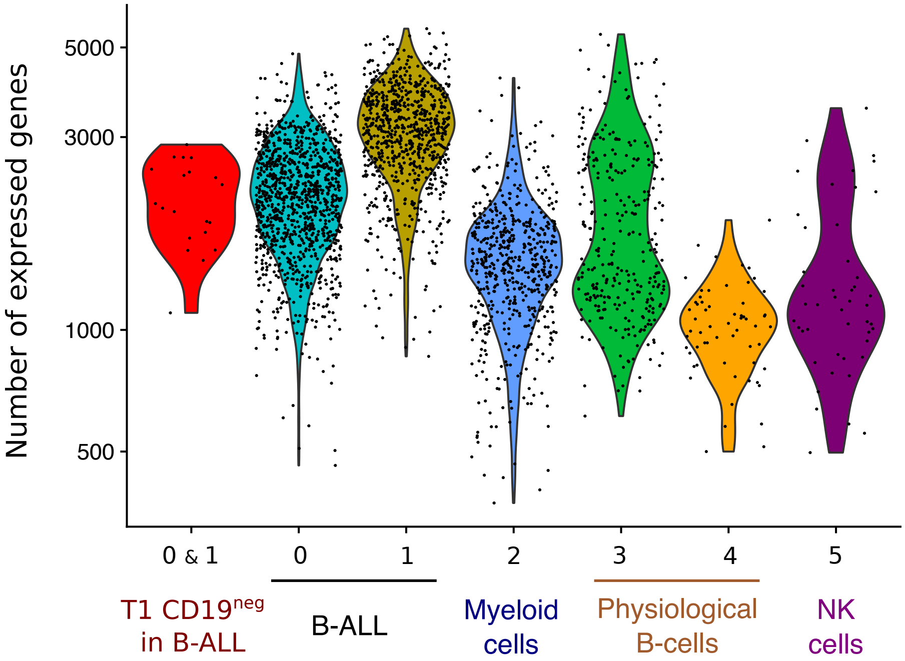

Supplementary Figure 10: Number of expressed genes per cell.
Violin plot showing the number of expressed genes in cells within the 6 clusters (0 to 5). Each dot represents a single cell. The 20 T1-CD19^{neg} cells found in clusters 0 & 1 were analyzed separately. Those T1-CD19^{neg} cells express sufficient genes (around 2090 genes/cell) to reliably assess their identity.

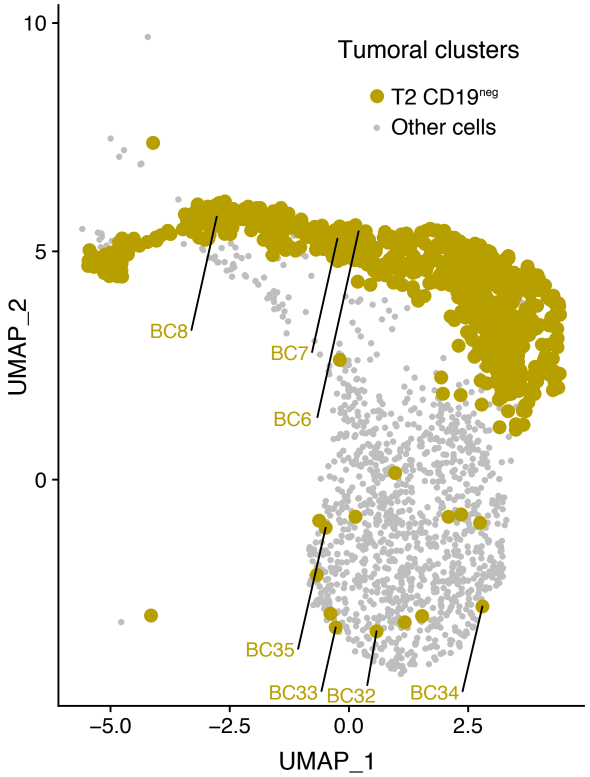

Supplementary Figure 11 (related to figure 2e): Localizations of backtracked T2-CD19^{neg} B-ALL cells.

UMAP plot focused on tumoral cells (clusters 0 and 1). T2-CD19^{neg} cells are highlighted in gold (all others cells are in grey). Cells identified by their BC number and tested by nested-PCR (Fig. 2e) are indicated. Cells with BC 6, 7 & 8 are localized in cluster 1, while cells with BC 32 to 35 are localized in cluster 0.
